# Work in New York

**Published:** 1930

**Authors:** 


### CHILD GUIDANCE.*

### I.

### Work in New York.

By Mrs. POSTHUMA, M.B., Ch.B.

In some marine insurance policies certain contingencies are grouped together under the term "acts of God." In former times children were looked upon in much the same way.

They were born (if Providence so willed), they were housed and fed more or less efficiently, they were educated more or less adequately (rather less than more in the case of girls), they were expected to be grateful to their parents for bringing them into the world, and to be the props of their old age.

These were the days when education according to the wise saw and the adage was the usual rule, "Spare the rod and spoil the child," "Little people should be seen and not heard," and the like. These precepts were evolved empirically through the ages, a good many, it may be suspected, rather designed for the comfort of the parent than the welfare of the child.

No doubt a good many children throve upon these principles or in spite of them, but there must have been always a large number to whom such generalizations must have been essentially useless or harmful. It is only comparatively recently that any general tendency has arisen to look upon the child as an individual, with the right to be considered as such from his first month

* Papers read at a meeting of the Bath and Bristol Branch of the British Medical Association held on 26th March, 1930.

onward. It has also come to be recognized that his environment, especially in the emotional sense, has a very large share, dating from his earliest days, in the process of his development.

The swing of the pendulum from the old, rigid, unimaginative methods of upbringing for a time produced a period of complete license in the nursery. Self-expression for the child was the slogan, and parents seemed to forget that "growing up" to adjust successfully and happily to the social restrictions and emotional complications of our present state of civilization is no such easy matter, and that it is their duty to help the young adventurers in a strange world, not by right of their parenthood, but by that of their wider experience and knowledge.

In the school world the growing recognition of the child as an individual can be traced by the rise of the school care committees and the school medical service.

In recent years the varying quality of minds has been taken into account, and the educational psychologist does valuable work in the grouping of children according to their intellectual equipment, and by giving advice on the question of vocational aptitudes. Most recently of all, the importance of the emotional element in the determination of the child's behaviour has been made the subject of study.

The present keen interest in these aspects of upbringing and education is met with in all parts of the civilized world, but it is in America that the development of this interest along practical lines has made most headway. The reason for this may be partly owing to the natural preoccupation of a young and growing country with its new generations, and partly owing to the fact that resources have not been crippled nor energies absorbed over there by the strain of the Great War, as inevitably is the case in those countries more closely involved in the catastrophe.

The child guidance movement, of which I propose to give you a brief account to-night, is the expression of the most recent endeavour to co-ordinate all these points of interest, physical, psychological, emotional and environmental, in the study of the child, and to formulate the results in such a way as to make a basis for scientific evaluation of the facts arrived at, the problem being to reach a working idea of why one individual fails to make an adjustment to a life which apparently presents no difficulty to another.

The Institute for Child Guidance in New York City, at which I had the good fortune to be working for a year, is the largest and most lavishly equipped in the country, being especially set up as a training centre. The permanent staff consists of the medical director, assistant director, three psychiatrists, three psychologists, a pediatrician, the chief of social service, nine fully-trained social workers, a statistician, and a large office staff.

In a newly-opened clinic the staff usually consists of the medical director, who does all the psychiatric work and gives the physical examinations as well, a psychologist, and a fully-trained social worker.

In New York last year there were six psychiatrists in training for a period of a year, five of them holding fellowships under the Commonwealth Fund and one under the Rockefeller Foundation; these were required to be fully-qualified men and women with (preferably) two years' special psychiatric experience as well. There were three psychologists in training also holding fellowships for a year.

There were about forty social workers doing a nine-months' period of training. This consisted of an excellent course of lectures and discussions at the New York School of Social Work under a psychiatrist, covering the field of normal and abnormal behaviour, with special bearing on the problems likely to be met with in child guidance work; they had various other lectures and demonstrations, and their practical experience in care work was gained at the Institute, where they worked directly under the trained staff of social workers.

For working purposes the Institute staff was divided into three units, each consisting of one psychiatrist, one psychologist and two social workers, to which were attached two psychiatrists, one psychologist and ten or twelve social workers in training.

The cases were sent up by the schools, private physicians or various social agencies.

The children varied in age from about a year up to eighteen years, and presented a vast variety of problems, varying from food-fads and temper-tantrums to truancy, lying, stealing and sex delinquencies.

A few of them were definitely psychotic, or at least showing symptoms with ominous suggestion; occasionally we had a feeble-minded child, but these were not accepted for treatment unless the circumstances were in some way unusual.

The majority of the children were average in intelligence, but quite a large proportion rated as of superior intelligence, and I had one boy who was referred by the principal of his school as being very unstable and in need of vocational guidance, who turned out to possess creative ability of a high order and an amazing talent for drawing.

One interesting fact may be noted, viz. that the vast majority of these problem children came from unsatisfactory homes, either foster homes, both parents being dead, or where the parents were divorced or on the verge of separation, or where one or both were alcoholic or drug addicts, or where the parents were merely unstable themselves or of an extra low order of intelligence and incapable of managing a home. It was impossible to take all cases referred, so the main particulars were obtained and were brought up at a referral committee, which consisted of a psychiatrist, a psychologist and three of the chief social workers. The case was discussed from the various angles, and the most urgent and suitable accepted for treatment.

This having been done, the four-fold attack, as Dr. Lowrey called it, was brought into action, which means that the case was studied equally from the four angles, (1) the environmental, (2) the physical, (3) the psychological, (4) the psychiatric.

The first step was the visit of the social worker to the family. Much emphasis was laid on the fact that treatment here really began on the first day of contact, as future success often depended on how effectively the point of view of the Institute was presented to the parents, and in consequence how much confidence they would place in our line of treatment, and how much co-operation we could expect from them.

The social worker in the course of one or more visits would gather the essential etiological facts of the problem with a more or less complete family history, special note being taken of any possibly hereditary disease, and of any emotional difficulties the parents may have encountered in their own childhood, and of any jealousy situations or favouritism in the present family group.

The patient was next brought to the Institute for a thorough physical examination by the pediatrician. This being completed, the psychological study was undertaken. In the hands of a psychologist trained in this sort of work a great deal of helpful information might be forthcoming, not only as establishing some reliable guide as to the child's intelligence, but also throwing valuable light on his powers of concentration, perseverance, methods of meeting his difficulties and handling his work ; emotional inhibitions might appear with important bearing on the case, and the existence of any special aptitude or disability be determined. I had one boy of eight who was sent up for nervousness, refusal to read in class, and crying when told to do so. He would read no story books, but had apparently no difficulty in getting what he wanted out of the popular scientific magazines. He tested out as having very superior intelligence with marked mechanical ability, but as possessing an equally marked reading disability. He could make out the meaning of a paragraph, but was unable to pronounce the words, and could not see why he should have to do so. The advisability of having a vocabulary if he wanted to get on well and go to college was put before him, and he saw the point. After three months' special coaching at the Institute he had quite caught up with the rest of the class and had become a voracious reader. The crying fits and nervousness had entirely disappeared, and he was improved in every way, having gained poise and self-assurance.

The psychiatrist's interview with the child was usually the last in the series of investigations, and the point here was to determine the patient's own reaction to the problem and the situation in general with the emotional factors at work. With the younger children it was a very informal affair. One played with the small person, and aimed at establishing a friendly and uncensorious relationship, in the course of which many interesting facts might come to light ; sometimes the picture thus gained would be surprisingly at variance with the reports gathered from the grown-ups connected with the child.

With the older boys and girls it was often found advisable for the psychiatrist to see them as soon as the case was referred ; it was important to gain their interest and co-operation, and to give them some understanding of the Institute methods before the social worker approached their families for information, which procedure they might naturally resent as a threat to their young independence.

When once the social history and the various examinations were completed, the initial conference was arranged, and we all met together to discuss the case from our various angles, and to decide on a plan of treatment. One of the medical directors was usually present, one or more of the chief social workers, and any physician, teacher or representative of outside agencies interested in the case might be invited to attend, if such a course were considered useful or advisable.

The treatment plan varied so much with every case that it is not easy to generalize.

With the younger children, *i.e.* up to about seven, the development of the symptomatic behaviour for which they were referred, temper-tantrums, food-fads and the like, could, in most instances, be traced back to a desire for attention, *i.e.* if they could not get what they felt was the rightful amount of notice from their parents in a desirable form they would set out to get it in an undesirable manner, seeming to prefer a constant series of scoldings and beatings to no attention at all.

Treatment in these cases lay not with the child itself so much as with the family. It was the social worker's business to interpret the situation to the parents, and to proffer suggestions on training and management in such a way as to be acceptable by them. She would pay them weekly, fortnightly or monthly visits according to the urgency of the case ; she worked in close co-operation with the psychiatrist and the psychologist by means of frequent informal conferences, and any new development was at once reported to us all.

Sometimes the main line of treatment lay with the psychological department ; in one case the child came up for coaching in reading while I had weekly interviews with the mother, a very interesting but most unstable person, with whose own personality as determined by unfortunate experiences lay the principal problem in the case.

With the older children and the adolescents the usual procedure was for the patient to come up for more or less frequent interviews with the psychiatrist, while the social worker kept in touch with the family.

In all our training much emphasis was laid on the importance of considering the situation as a whole, and of realizing the interaction of all the personal and environmental factors on the problem as presented. The social worker was encouraged to discuss the cases freely with the psychiatrist and the psychologist, and the value of the whole method would seem to lie largely in the excellence of the team work thus obtained ; the brunt of the treatment might fall sometimes on the psychiatrist, sometimes on the psychologist, sometimes on the social worker, but by this system of close co-operation we were enabled to keep sight of the fact that each case should always be viewed from the three angles, and were able to give the underlying mechanisms their full evaluation as further study might bring them to light.

It is useless to think that the Child Guidance movement is out to put an end to juvenile delinquency or to prevent the development of adolescent psychoses ; it may, however, be taken as probable that a certain proportion of the cases which are being treated, and which are apparently progressing satisfactorily, would, if left to themselves, grow up into those unhappy, ill-adjusted, neurotic semi-failures in life who are a misery to themselves and their families, a type met with only too commonly in all countries. The work has not been going on for long enough to show end-results, but as we see very clearly the unhappiness of the parental childhood experiences being perpetuated (often unconsciously) in the treatment of the child, it would seem not unreasonable to hope that the increased sense of security and consequent happiness brought into the lives of these children by the sympathetic study of their problems may bear fruit not only now but in the next generation.

In the United States Child Guidance Clinics are now springing up all over the country in response to a widespread demand.

Through the generosity of the Commonwealth Fund of America a demonstration clinic has been started in Islington, London ; the same organization has been inaugurating clinics in various large cities in the States, which are now supporting themselves.

It is sincerely to be hoped in the interests of the rising generation that this public-spirited lead may be followed in this country with an equal degree of enthusiasm and success.

